# Does the mismatch negativity operate on a consciously accessible memory trace?

**DOI:** 10.1126/sciadv.1500677

**Published:** 2015-11-13

**Authors:** Andrew R. Dykstra, Alexander Gutschalk

**Affiliations:** Department of Neurology, Ruprecht-Karls-Universität Heidelberg, 69120 Heidelberg, Germany.

**Keywords:** consciousness, awareness, Perception, short-term memory, mismatch negativity, auditory cortex, magnetoencephalography, ERP, ERF

## Abstract

The extent to which the contents of short-term memory are consciously accessible is a fundamental question of cognitive science. In audition, short-term memory is often studied via the mismatch negativity (MMN), a change-related component of the auditory evoked response that is elicited by violations of otherwise regular stimulus sequences. The prevailing functional view of the MMN is that it operates on preattentive and even preconscious stimulus representations. We directly examined the preconscious notion of the MMN using informational masking and magnetoencephalography. Spectrally isolated and otherwise suprathreshold auditory oddball sequences were occasionally random rendered inaudible by embedding them in random multitone masker “clouds.” Despite identical stimulation/task contexts and a clear representation of all stimuli in auditory cortex, MMN was only observed when the preceding regularity (that is, the standard stream) was consciously perceived. The results call into question the preconscious interpretation of MMN and raise the possibility that it might index partial awareness in the absence of overt behavior.

## Teaser

A change-related component of the auditory evoked response long thought to be preattentive and preconscious may actually require consciousness.

## INTRODUCTION

Short-term memory is a fundamental construct in cognitive science, where one of the central questions is whether and to what extent its contents are consciously accessible (*1*–*3*). In audition, the neural basis of short-term memory is often studied via mismatch negativity (MMN) (*4*–*6*), a change-related component of auditory evoked response and one of the most oft-studied brain responses in neuroscience. Normally elicited by a deviant stimulus in the so-called auditory oddball paradigm (*6*), MMN can be observed in response to any discriminable violation of an otherwise regular stimulus sequence (*7*).

Although its sensitivity to task and stimulation contexts is well established (*8*), the prevailing functional view of MMN is that it reflects automatic change detection, operating on a preattentive and even preconscious representation of the preceding context regularity (that is, the standard stream) (*7*). On the basis of this interpretation, MMN has been used extensively to assess the neural basis of preconscious auditory memory. Apart from the many studies claiming that it is only mildly sensitive to manipulations of directed attention, the preconscious view of MMN also stems from the fact that it has sometimes been observed in various states of behavioral unconsciousness (for example, sleep, sedation, and disorders of consciousness) (*9*, *10*). However, these studies have failed to reach a consensus on whether MMN can be elicited in the absence of perceptual awareness of the standard stream, and to our knowledge, this has never been directly examined in the healthy, waking brain.

**Fig. 1:**
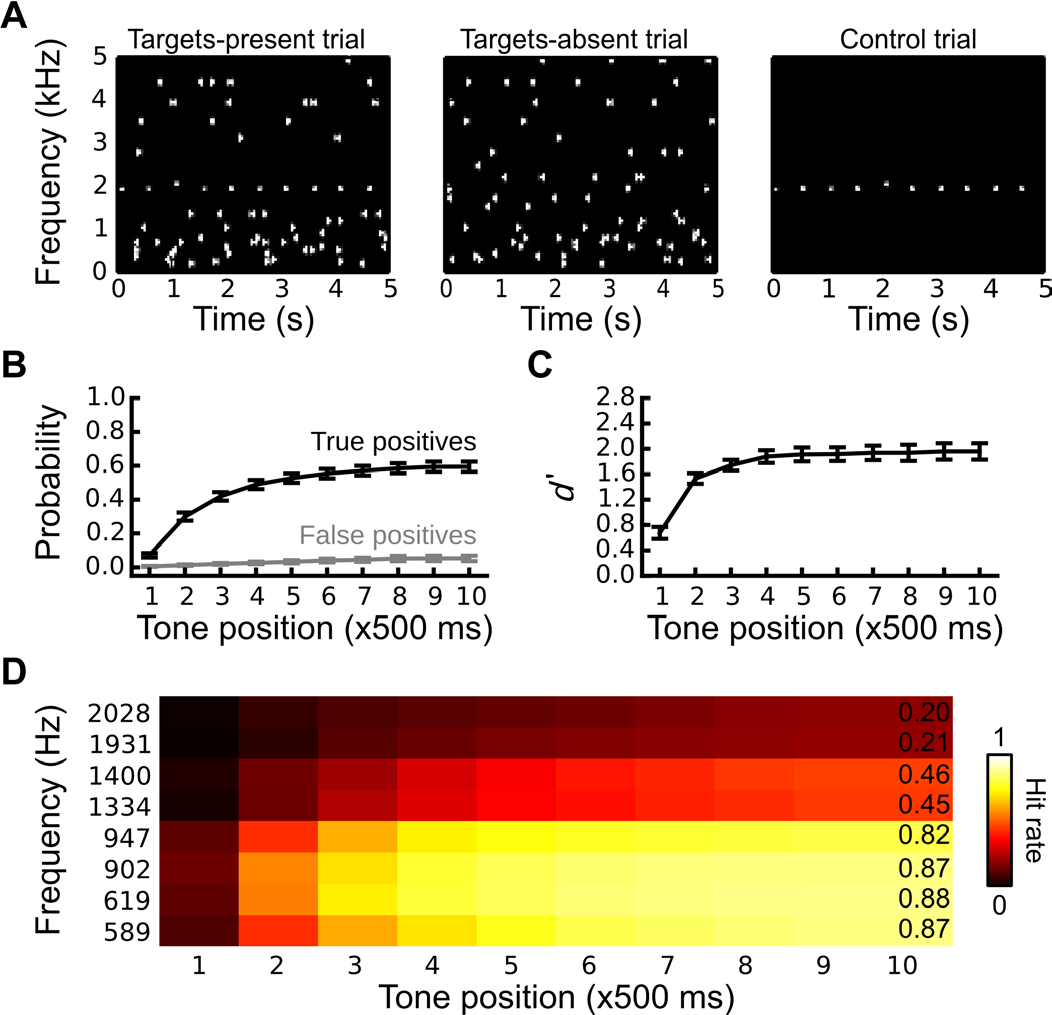
Stimulation paradigm and behavioral results. (**A**) Spectrographic representation of a targets-present masked trial (left), a targets-absent masked trial (middle), and an unmasked control trial (right). (**B**) True- and false-positive rates (that is, hits and false alarms) as a function of position within the stimulus sequence. (**C**) Corresponding *d*′. (**D**) True-positive rates as a function of position, binned by target frequency.

The present study addressed this question using informational masking (*11*) and simultaneous magnetoencephalography (MEG). Classical auditory oddball sequences were embedded in random multitone masker “clouds” (Fig. 1A and audio S1 and S2), rendering them sometimes inaudible despite spectral isolation and suprathreshold sensation levels (*12*). In each trial, listeners (*N* = 20) indicated via button press the moment at which they began to hear out the regular standard-stream sequence from the irregular multitone background, allowing us to bin MEG responses according to listeners’ moment-by-moment perception of the standard stream. Sequences consisting only of the multitone cloud stimulus (“catch trials”) facilitated the measurement of false-positive rates and the sensitivity index *d*′. Listeners were instructed to ignore the pitch change associated with the frequency deviant. Thus, in contrast to previous work examining the task dependence and context dependence of MMN, our paradigm required no stimulus or task manipulations to produce different perceptual interpretations of the deviant-preceding standard stream.

## RESULTS

### Listeners were sensitive to the presence of oddball sequences

On average, only 59% of all standard streams were perceived when they were embedded in multitone masker clouds (Fig. 1B). Listeners were sensitive to the presence of such sequences, however, as the false-positive rate never exceeded 7%, on average, resulting in a sensitivity index (*d*′) that plateaued around 2 (Fig. 1C). Repeated-measures analysis of variance (ANOVA) confirmed significant main effects of response (true-positive versus false-positive rates, *F*_1,19_ = 259.8, *P* < 0.0001), tone position (*F*_1,19_ = 212.7, *P* < 0.0001), and their interaction (*F*_1,19_ = 129.6, *P* < 0.0001), indicating a larger difference between true-positive and false-positive rates for later tone positions. Sorting the true-positive rates by standard-stream frequency further revealed that lower-frequency standard streams were easier to detect, especially for later tone positions (Fig. 1D) (main effect of frequency: *F*_1,19_ = 106.4, *P* < 0.0001; main effect of tone position: *F*_1,19_ = 237.1, *P* < 0.0001; two-way interaction between frequency and tone position: *F*_1,19_ = 56.0, *P* < 0.0001).

### MMN was not generated in the context of unperceived standard streams

**Fig. 2:**
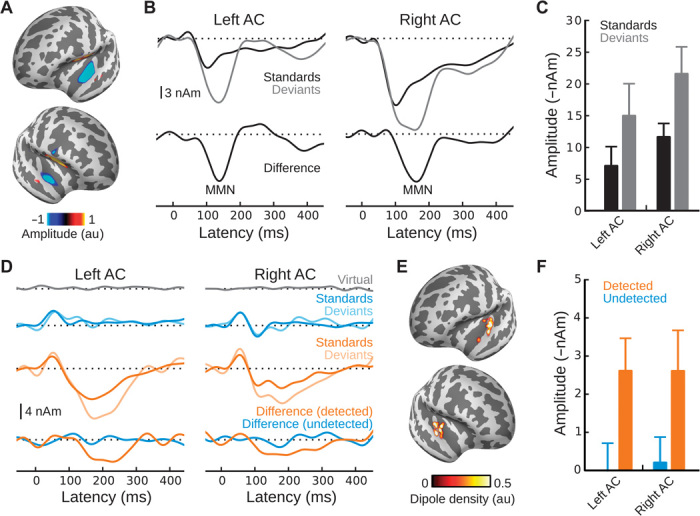
Neural activity in the control (A to C) and masked (D to F) conditions. (A) Grand-average normalized dSPM between 100 and 150 ms for deviants minus standards under control (unmasked) conditions. (B) Grand-average MxNE solutions for (top) standards (black traces) and deviants (gray traces) and (bottom) their respective difference waveforms. (C) Quantified amplitudes for standards (black) and deviants (gray). (D) Grand-average MxNE solutions for virtual targets (gray), deviants and standards for undetected standard streams (blue), deviants and standards for detected standard streams (orange), and their respective difference waveforms (deviants minus standards). (E) Corresponding dipole locations. (F) Quantified amplitudes for MMN responses generated in the context of detected (orange) and undetected (blue) standard streams. au, arbitrary units.

To examine whether perceptual awareness of the standard stream is required for MMN generation, we first verified that MMN could be elicited by our oddball paradigm in the absence of the multitone masker. Indeed, in quiet, deviants elicited larger peak amplitudes than standards in both left and right auditory cortex (AC) on the anterior superior temporal plane (aSTP) (Fig. 2, A to C) (deviants versus standards: *F*_1,19_ = 19.5, *P* < 0.0005).

For oddball sequences embedded in multitone masker clouds, MEG responses to both deviant and standard stimuli were binned and averaged separately according to the context in which they occurred (that is, perceived or unperceived standard streams) (Fig. 2, D to F). Additional epochs, composed of the MEG response to “virtual targets,” were created from the catch trial (that is, masker-alone) stimuli by time-locking the MEG signal relative to the potential positions of target tones, had they been present. This condition serves as a baseline and should result in a flat average response due to the random nature of the time-locking, thus yielding an empirical estimate of the signal-to-noise ratio of the averaging procedure (Fig. 2D, gray traces).

Examining the source waveforms from this analysis, it is immediately apparent that only deviants occurring in the context of perceived standard streams elicited an MMN (Fig. 2D, orange and blue traces) located on the aSTP (Fig. 2E). A three-way ANOVA performed on quantified amplitudes (Fig. 2F), with deviance (deviants versus standards), percept (detected versus undetected), and hemisphere (left versus right) as factors, revealed significant main effects of deviance (*F*_1,19_ = 11.4, *P* < 0.005) and percept (*F*_1,19_ = 29.5, *P* < 0.0005), as well as a significant two-way interaction between them (*F*_1,19_ = 5.7, *P* < 0.05), suggesting that deviants elicited larger responses than standards, but only if they occurred during perceived standard streams. This was confirmed by two subsequent two-way ANOVAs (each with deviance and hemisphere as factors) for amplitudes in the context of (i) undetected standard streams (where there was no significant effect of deviance) and (ii) detected standard streams (where there was a significant effect of deviance) [undetected: *F*_1,19_ = 0.0, not significant (n.s.); detected: *F*_1,19_ = 13.9, *P* < 0.005], as well as a direct comparison of MMN amplitude from deviant-standard subtraction waveforms (detected versus undetected: *F*_1,19_ = 5.7, *P* < 0.05; no significant effect of hemisphere; no significant interaction).

A supplemental analysis controlling for the frequency bias in the behavioral data (cf. Fig. 1D) produced the same pattern of results (fig. S1). In addition, the per-frequency deviance responses in the control conditions were nearly identical (fig. S2). Thus, the difference between detected and undetected deviance responses cannot be accounted for by differing distributions of higher-frequency versus lower-frequency stimuli comprising the detected and undetected bins.

To further examine whether MMN may have been elicited during unperceived standard streams, we conducted a Bayes factor analysis, which provides an indication of whether nonsignificant results actually support the null hypothesis (Bayes factors ≤ ^1^/_3_, by convention) versus being merely insensitive to differences between groups (Bayes factors around 1) (cf. Materials and Methods) (*13*). Assuming a uniform distribution of plausible population effect sizes with the maximum taken as the mean MMN amplitude elicited in the context of perceived standard streams, we obtained a Bayes factor of 0.29 (0.27 after controlling for the aforementioned frequency bias). Even when using a more conservative half-normal effect-size plausibility distribution, we still obtained values of 0.42 and 0.37 for the uncontrolled and controlled analyses, respectively. Thus, rather than merely being insensitive to its potential presence, our data provide evidence that no MMN was generated in the context of unperceived standard streams.

### All stimuli were represented in primary AC

**Fig. 3:**
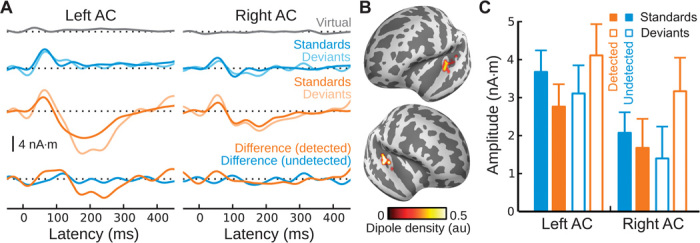
Neural activity under masked conditions, with emphasis on the P1 latency range. (**A**) Grand-average MxNE solutions for virtual targets (gray), deviants and standards for undetected standard streams (blue), deviants and standards for detected standard streams (orange), and their respective difference waveforms. (**B**) Corresponding dipole locations. (**C**) Quantified amplitudes for P1 responses generated in the context of detected (orange) and undetected (blue) standard streams, for both standards (left) and deviants (right).

The lack of an MMN during unperceived standard streams raises the question of whether unperceived standards were at all represented at the cortical level. We therefore carried out an additional source analysis focused on early activity, which indicated that all stimuli generated a P1 response (already visible in the MMN-focused analysis of Fig. 2D) irrespective of listeners’ awareness (Fig. 3 and table S1). Thus, all stimuli were represented in cortex at an early stage of processing. Like the MMN, the P1 was also significantly larger for deviants that occurred in the context of perceived standard streams, but not unperceived standard streams. This was confirmed by a two-way interaction between deviance and percept (*F*_1,19_ = 10.3, *P* < 0.005; no significant main effects), as well as by a significant effect of deviance in the context of detected standard streams (*F*_1,19_ = 6.9, *P* < 0.05), but no such effect in the context of undetected standard streams (*F*_1,19_ = 1.8, n.s.). The same pattern of results held after epoch equalization was applied, although the comparisons did not reach statistical significance (fig. S3).

### Masker tones elicited similar activity before and after standard-stream detection

**Fig. 4:**
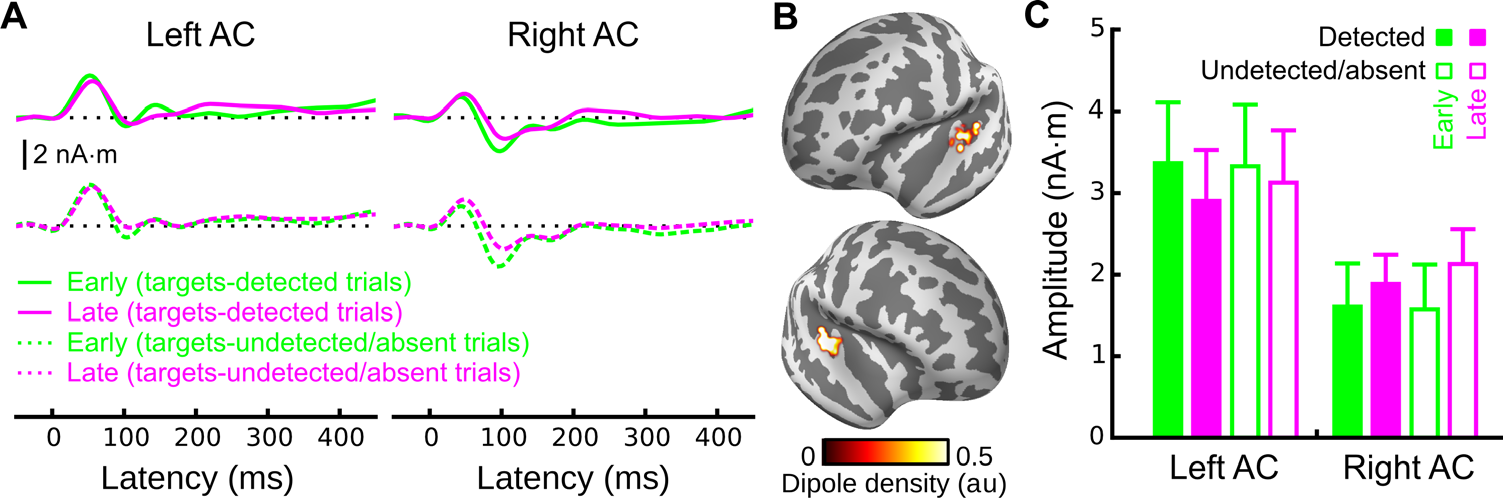
Neural activity elicited by masker tones, with emphasis on the P1 latency range. (**A**) Grand-average MxNE solutions for responses elicited by masker tones for targets-detected trials (solid traces; top) and targets-undetected/targets-absent trials (dotted traces; bottom) for early (green) and late (magenta) time intervals. Epochs for targets-detected trials were created by splitting those trials into two time intervals (before and after target detection). Epochs for targets-undetected/targets-absent trials were created by splitting those trials into two time periods based on the average detection time of the targets-detected trials. (**B**) Corresponding dipole locations. (**C**) Quantified P1 amplitudes in response to masker tones both before (green) and after (purple) detection of the standard stream. Closed (open) bars are for targets-detected (targets-undetected) trials. A three-way ANOVA with hemisphere, target detection, and time interval as factors revealed significant two-way interactions between hemisphere and interval (*F*_1,19_ = 9.2, *P* < 0.01) as well as a marginally significant two-way interaction between target detection and interval (*F*_1,19_ = 3.3, *P* = 0.09). However, subsequent paired comparisons did not show significant effects of the time interval on either left AC or right AC in the direction one would expect based on an attentional account of the data (table S2). A similar pattern of results was observed when the more anterior MMN-defined source space was used (cf. figs. S4 and S6).

To further evaluate a potential attentional interpretation of our findings, we carried out an additional analysis on neural responses to masker tones. If the difference in the responses observed in the context of detected versus undetected standard streams were the result of selective attention to the standard stream after its detection, we would expect to observe correspondingly weaker responses to masker tones after listeners became aware of the standard stream. Although there was a trend for masker tone P1 responses to be smaller after target-stream detection in left AC, this effect was small and not present in right AC (Fig. 4 and table S2; see also fig. S4). Furthermore, we observed similar effects when masker-evoked responses were compared between the early portions and the late portions of the trials for which target streams were either absent or present but subliminal, suggesting that the effect is likely related to adaptation rather than attention. Finally, an attention-based account of our findings would also predict enhanced P1 responses for detected versus undetected standards. However, if anything, we observed the opposite (that is, smaller P1 responses for detected versus undetected standards) (Fig. 3C) (left AC: *T*_19_ = 2.1, *P* < 0.05; right AC: *T*_19_ = 0.82, n.s.).

**Fig. 5:**
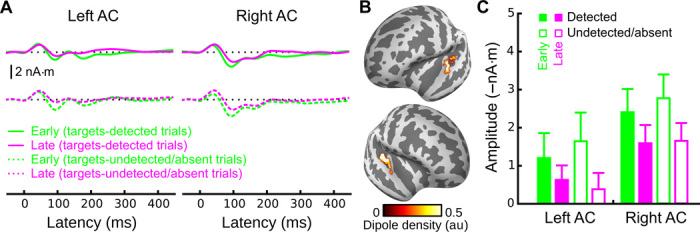
Neural activity elicited by masker tones, with emphasis on the N1 latency range. (**A**) Grand-average MxNE solutions. (**B**) Corresponding dipole locations. (**C**) Quantified N1 amplitudes. A three-way ANOVA with hemisphere, target detection, and time interval as factors revealed a significant main effect of interval (*F*_1,19_ = 15.1, *P* < 0.005) as well as a significant two-way interaction between interval and target detection (*F*_1,19_ = 5.1, *P* < 0.05). However, this interaction went in the opposite direction that would be expected based on an attentional account of the data (that is, the early-late difference was larger for targets-undetected and targets-absent trials; table S2). Here, the source space used for the masker-elicited N1 was the same as for the masker-elicited P1. The same pattern of results was found when the more anterior MMN-defined source space was used (cf. figs. S5 and S6).

Finally, in addition to P1, masker tones also elicited a small but reliable N1, which is generally much more sensitive to attention than P1 (*14*). We thus performed a similar masker-tone analysis focused on the N1 (Fig. 5 and table S2; see also fig. S5). Here, the before/after effect present in targets-detected trials (that is, smaller masker-elicited N1 responses after target detection) was even larger for targets-undetected and targets-absent trials, the opposite of what would be expected based on an attentional suppression of masker-tone responses after target detection. These data are thus strong evidence against an attentional interpretation of our data and suggest that the reduction in amplitude observed for after-detection versus before-detection masker responses (for both the P1 and the N1) was the result of adaptation rather than a shift of selective attention.

## DISCUSSION

Our findings have several important implications for the interpretation of MMN, its neural generators, and its diagnostic utility in disorders of consciousness. Functionally, they suggest that MMN operates on conscious stimulus representations rather than preconscious ones (*7*), consistent with a recent interpretation (*8*) emphasizing its sensitivity to auditory perceptual organization, which, in our paradigm, likely determined listeners’ perception of the standard stream. However, in contrast to previous studies, our paradigm required no task or stimulus differences to manipulate MMN. This interpretation does not necessarily contradict previous work showing that behavioral detection of deviance may not be necessary for MMN generation (*15*). However, others have shown a strong correspondence between MMN latency and behavioral reaction time (*16*), highlighting MMN’s proximity to conscious processes. Nor does our interpretation argue against the existence of preconscious auditory memory traces (*17*), only that such traces may be insufficient for the MMN process (*18*).

An alternative interpretation that cannot be ruled out relates to attention. Dissociating awareness from attention is certainly challenging (*2*, *19*). Here, because hearing the standard stream makes it easier to attend, attention—and not awareness—might have been the crucial factor underlying MMN generation. Although this interpretation is attractive given that (i) attention can modulate MMN (*20*–*24*) and (ii) general attention to the stimuli used here was undoubtedly required for listeners to perform their detection task, we find it unlikely for several reasons.

First, the results of our masker-tone analysis strongly argue against an attentional interpretation. Second, several studies have observed MMN when participants actively ignored the stimuli by engaging in other, sometimes highly demanding tasks (*7*, *25*). In these studies, basic perception of the standard stream is nevertheless likely given the otherwise quiet conditions. Third, in contrast to previous studies examining attentional modulation of MMN, the deviant tones used here were never task-relevant; thus, although listeners could have attended to the standard streams after becoming aware of them, they were neither required nor instructed to do so. Last, as we detail below, MMN can sometimes be observed during states of behavioral unconsciousness (that is, sleep, sedation, and minimally conscious state), where basic awareness of sensory stimuli seems more likely than the ability to attend them.

Thus, the strong attentional effects observed by previous MMN studies may have instead been a result of stimuli/task manipulations inducing changes in awareness. However, a recent study using perceptually bistable triplet sequences did not find such effects (*26*), perhaps because the deviant stimulus used was deviant regardless of perceptual organization.

Anatomically, our results agree with previous work by Näätänen and Alho (*27*) suggesting that the aSTP is the predominant MMN generator. First, it was clearly the most active area in our source analysis. Second, only deviants embedded in detected standard streams elicited an MMN, and previous work has implicated nonprimary AC in auditory perceptual awareness (*28*). Finally, all stimuli elicited earlier responses (P1) thought to arise from primary AC (*29*), and although intracortical data from humans (*30*), cats (*31*), and nonhuman primates (*32*) have identified an MMN-like response there, our source analyses did not show deviance-related activity in this area at MMN latency.

In addition to temporal generators, some studies have reported a frontal component of MMN (*33*), consistent with diminished MMN amplitudes after frontal lesions (*34*) and modeling work (*35*) [but see Wacongne *et al*. (*36*)]. There was no evidence for such a component in our data, suggesting either that no frontal activity was evoked by our paradigm or that MEG may not be sensitive to frontal MMN sources (*37*). This does not rule out that frontal processes, even if they do not contribute to MMN per se, might constitute a necessary antecedent to its generation. To the extent that frontal cortex is necessary for conscious access (*38*), this would be consistent with our finding that MMN generation required perceptual awareness of the standard stream.

Although we did not observe a deviance effect in primary AC at the time of MMN, our data did show an earlier effect around the P1 latency, consistent with aforementioned animal models and recent human studies (*39*). Like MMN, this effect was observed only in the context of perceived standard streams, a somewhat surprising result in light of the present and previous results showing that the presence or absence of P1 does not covary with perceptual awareness (*40*). If the P1 deviance effect we observed is related to stimulus-specific adaptation (SSA), as has been observed in other contexts (*41*, *42*) and proposed for MMN (*43*, *44*), then such SSA might be highly context-dependent—even at early stages of cortical processing—possibly as a result of perceptually gated tuning-curve sharpening (*45*, *46*).

However, a recent study observed an early (~85 ms) deviance effect that persisted in deep sleep (*47*), an apparent discrepancy with our finding that this early deviance response (~55 ms in our data) was abolished during unperceived standard streams. The reasons for this are unclear but may be related to the tuning-curve hypothesis outlined above (that is, when subliminal, standards and deviants activated similar neuronal populations). Here, it is worth noting that Straus and colleagues (*47*) did not observe this early deviance response for “global” deviants (that is, stimuli that were physically identical to the immediately preceding context but deviant from the standard pattern on that block) (*48*). Another possibility is substantial paradigmatic difference (vowels versus tones, short versus long stimulus onset asynchrony, no masker). Both arguments would predict that the P1-related deviance effect we observed could appear in the context of unperceived standard streams given appropriate stimulus parameters.

A major difference between typical MMN paradigms and that used here is the complexity of the stimuli. Whereas the canonical MMN paradigm involves only a single stream of tones (frequent standards and rare deviants, as in our control conditions), the multitone masker presents listeners with a perceptually demanding segregation task and probably led to the slightly later/broader MMN we observed in masked versus control conditions. Specifically, the multitone masker likely decreased the salience of the difference between deviants and standards, a factor known to alter MMN morphology and peak latency (*15*, *16*).

Whether generation of MMN requires standard-stream awareness in simpler stimulus configurations is debatable. However, in quiet, any oddball sequence for which MMN can be elicited will nearly always be perceived, and devising a paradigm in which oddball sequences are consciously accessible yet sometimes subliminal is challenging, and a primary advantage of the paradigm used here. Another way that this has been examined is through the recording of evoked responses during various states of behavioral unconsciousness, including sleep (*49*, *50*), sedation (*51*, *52*), and disorders of consciousness (*10*), where it is often concluded that MMN can be observed in the absence of consciousness, defined behaviorally (*9*). As mentioned in the Introduction, however, these studies have failed to reach a consensus on the relationship between perceptual awareness and MMN.

During sleep, MMN is typically observed only in particular stages [for example, rapid eye movement (REM) sleep or light stage 1] where individuals can be partially aware of—even behaviorally responsive to—external stimuli (*47*, *53*–*55*) and is completely abolished in deeper stages except for hypersalient, very rare, or extremely deviant stimuli (*56*). Some studies have failed to observe MMN even in REM sleep (*57*, *58*). A very recent study has confirmed this general pattern (*47*) and, given that MMN was the only component to be completely abolished in deep sleep (in fact, before that, in nonresponsive stage 1), is consistent with the notion that MMN could reflect partial awareness. The results are clearer in the anesthesia literature, where MMN can be observed under sedation (*51*, *52*) but with markedly reduced amplitudes; it is abolished during (*59*) or even before (*60*) behavioral unconsciousness. The notion that MMN is linked to perceptual awareness is also supported by studies showing its sensitivity to violations of syntax (*25*, *61*), which has been shown to depend on listeners’ level of consciousness (*62*).

Perhaps most controversial is the literature on disorders of consciousness, where many studies have observed MMN during behavioral unconsciousness. Crucially, however, the presence of an MMN is highly predictive of imminent recovery in such patients (*10*, *63*, *64*), even though they are not generally thought to be even partially aware at the time of its elicitation (*9*). Our findings suggest arriving at this conclusion with caution and raise the possibility that the presence of MMN, or its emergence across recording sessions (*65*), could be one of the earliest available indicators of partial awareness.

## MATERIALS AND METHODS

### Ethics statement

All procedures were approved by the Institutional Review Board at the University Hospital Heidelberg. Each participant provided written informed consent before participation.

### Listeners

Twenty healthy participants (female, 11; male, 9) aged between 21 and 32 years (mean ± SD, 26 ± 4 years) took part in the study. None of the listeners reported any history of hearing or neurological disorders. Seven additional subjects were excluded from the analysis because of poor behavioral performance (*d*′ < 1 or false-alarm rate > 0.3) (*n* = 3) or inability to obtain anatomical magnetic resonance imaging (MRI) (*n* = 4).

### Stimuli and procedure

Sound stimuli were auditory oddball sequences (~5 s long) consisting of 10 short-duration pure tones (100 ms), with 10-ms on-ramps and off-ramps and 500-ms stimulus onset asynchrony. Only one deviant tone was presented in each trial and could occur anywhere between the third position and the eighth position within the sequence (inclusive). Four blocks of sequences (trials) were presented. The first and last blocks served as controls in which only oddball sequences (*N* = 120) were presented. Standard (deviant) tone frequencies were 589 (619), 902 (947), 1334 (1400), and 1931 (2028) Hz, reversed in half of the trials.

Listeners were asked to listen to the sequences and were required to initiate each trial with a button press. In addition to keeping the subjects alert during the measurement, the requirement that they initiate each trial also allowed them brief rest periods during which they were allowed to blink or close their eyes. In the second and third blocks, the same oddball sequences used in blocks 1 and 4 were embedded in a complex multitone masker consisting of tones randomly placed in time and frequency (*12*, *66*). Individual masker tones were drawn from a log-uniform distribution between 200 and 5000 Hz (specifically 0.2, 0.25, 0.30, 0.36, 0.43, 0.51, 0.59, 0.68, 0.79, 0.90, 1.03, 1.17, 1.33, 1.51, 1.71, 1.93, 2.18, 2.45, 2.76, 3.10, 3.48, 3.90, 4.37, and 4.89 kHz). Listeners’ tasks on these blocks were to (i) indicate via button press the moment at which they began to hear out a regularly repeating target-tone stream amidst the multitone masker and (ii) initiate the start of the next trial. For the measurement of behavioral sensitivity (*d*′) to oddball sequences when embedded in multitone masker clouds, an oddball sequence was presented in only 80% (*n* = 120) of all trials (*N* = 150) in each masked block; the other 20% of the trials (*n* = 30) were “catch” trials in which no oddball sequence was presented. This enabled us to estimate false-positive rates and corresponding *d*′ values (*z*-transformed true-positive rates minus *z*-transformed false-positive rates) (*67*).

All sound stimuli were generated in MATLAB (The MathWorks) and stored as 32-bit .wav files with a sampling rate of 48 kHz. Digital sound files were converted into analog waveforms by an on-board sound card (RME DIGI96/8 PAD) and a freestanding digital-to-analog converter (RME ADI-8 DS; RME), which in turn were controlled by SoundMexPro software (SoundMexPro) in the MATLAB environment. The analog stimuli passed through a programmable attenuator (TDT PA5; Tucker-Davis Technologies) before being amplified (TDT HB7; Tucker-Davis Technologies) and presented to listeners via ER-3 insert earphones (Etymotic Research) at a comfortable listening level. Listeners’ behavioral responses were recorded via an optical button device from Current Designs Inc.

### Data acquisition and preprocessing

MEG was recorded at a sampling rate of 500 Hz (using a 160-Hz online low-pass filter) via a Neuromag-122 system with 2 orthogonal planar gradiometers at each of 61 locations around the head. Structural MRI scans with the same field of view and resolution (1-mm isotropic), including T1-weighted magnetization-prepared rapid gradient echo (MPRAGE) and multiecho fast low-angle shot (FLASH) sequences, were acquired for each subject using a 3-T Siemens TIM Trio scanner.

Raw MEG data from each block were visually inspected to identify and reject time epochs or channels containing large artifacts or flat signals. They were then bandpass-filtered between 0.5 and 15 Hz and cleaned of blink artifacts using signal-space projection (*68*) in the MNE software package (http://martinos.org/mne) (*69*). The data were then epoched between −50 and 450 ms peristimulus (rejecting any epoch with gradiometer signals larger than ±10 pT/cm) and binned according to (i) standards versus deviants, (ii) standard/deviant target-stream frequency, and (iii) whether the standard stream was perceived before the occurrence of the deviant (in the case of masked conditions). Additional epochs, composed of virtual targets, were created from the catch trial (that is, masker-alone) stimuli by time-locking the MEG signal relative to virtual target tones (that is, the positions in the sequence of potential target tones, had they been present). Because the time-locking used here is effectively random, the resulting average response should be flat, yielding an empirical estimate of the signal-to-noise ratio of the averaging procedure.

For unmasked control blocks, the number of standard and deviant epochs was equalized across the four frequency bins before (i) averaging within bins and (ii) combining all standards and deviants into their respective bins to obtain average event-related fields for both standards and deviants. In our main analysis of masked conditions, epoch equalization was not performed because of the large effect of frequency on the detection of target streams (cf. Fig. 1D) and the resulting impact that such equalization would have on the signal-to-noise ratio of the responses. However, we did perform such epoch equalization as part of a supplemental analysis, as follows. Within each subject and for each frequency bin (F1, F2, F3, and F4), the numbers of epochs in the detected/undetected bins were equalized (separately for standards and deviants) by discarding trials in the bin with the higher initial number of epochs. Such equalization yields identical weighting of frequency bins for detected and undetected epoch distributions.

In addition to the analysis of target tones described above, we conducted a separate analysis of responses to masker tones. Individual masker-tone events were obtained by computing spectrograms of each stimulus sequence and finding energy onsets in each frequency band, after filtering out the band including the target tones (when present). Specifically, spectrograms were computed at each of the 24 frequencies in the log-normal distribution of possible masker-tone frequencies (plus intermediate frequencies) using 768-sample Hamming windows with an 87.5% overlap, yielding a temporal resolution of 2 ms, the same as for the 500-Hz MEG acquisition. This ensured that the timing of masker-tone onsets obtained using this method was no less accurate than if we had used trigger pulses recorded by the MEG acquisition system. Then, at each of the 24 possible masker-tone frequencies, we used an onset-detection algorithm that identified the first sample that exceeded 75% of the maximum power at the given frequency, ignoring the subsequent 100 samples at that frequency. This 100-sample (or 200-ms) “refractory period” likely resulted in some masker-tone events being missed, but this is unlikely to have affected the results given that there were 18,600 possible masker events across the two blocks [2 blocks × (120 targets-present trials/block × 10 500-ms windows/trial × 6 masker tones/window + 30 targets-absent trials/block × 10 500-ms windows/trial × 7 masker tones/window)].

The spectrograms were divided into two groups: (i) targets present and detected (targets-detected trials) and (ii) targets either absent or present but undetected (targets-absent and targets-undetected trials). Masker epochs (from −50 to 450 ms peristimulus) from these two categories were then subdivided into “early” and “late” epochs. For targets-detected trials, this corresponded to before and after target detection. For targets-undetected trials, this corresponded to before and after the average detection time from targets-detected trials. For targets-detected (targets-undetected) trials, this resulted in an average of 2107 (2361) and 5727 (6569) masker-tone events in the early and late time windows, respectively. The overall larger number of masker-tone events in targets-undetected trials reflects the fact that, when a target stream was present, it replaced one of the masker tones in each time window to preserve the overall stimulus energy across time. This analysis allowed us to examine whether the responses to masker elements changed as a function of target detection while controlling for potential effects of time.

With the FreeSurfer (http://surfer.nmr.mgh.harvard.edu) and MNE software packages, the MRI data were used to construct individualized, cortically constrained source spaces and boundary element models (BEMs). The high-resolution MPRAGE data were used to create three-dimensional (3D) reconstructions of the white and pial surface of each participant using FreeSurfer (*70*, *71*). The resulting white matter surface was decimated (to about 4100 dipole locations per hemisphere) to create the MEG source space. The resulting dipoles represent, on average, a cortical surface area of about 24 mm^2^, with an intersource spacing of about 5 mm. The FLASH images were used to produce high-resolution inner-skull, outer-skull, and scalp surfaces. These, in turn, were decimated and used to construct participant-specific three-layer BEMs. The MEG and MRI coordinate frames were aligned by manually demarking the fiducial points on the high-resolution scalp surface. Refinement of this initial 3D affine registration was carried out using the iterative closest-point algorithm (*72*) as implemented by the MNE suite.

### Source analysis

We used a combination of anatomically constrained, noise-normalized, minimum-norm estimates (*73*, *74*) known as dynamic statistical parametric mapping (dSPM) (*75*) and sparse inverse estimates based on an L1/L2 mixed norm (MxNE) (*76*). For both masked and control conditions, we first computed the dSPM for the deviants-minus-standards subtraction condition. The resulting masked dSPMs served as priors in the computation of MxNE solutions, which proceeded as follows.

First, because this analysis indicated that the primary—indeed only—generator of MMN in our data was located on the superior temporal plane anterior to the transverse temporal gyrus (TTG) (cf. Fig. 2A), we restricted the source space for the MMN component by explicitly zeroing out vertices that were not in this area during sparse source analysis (cf. fig. S5). This was performed to avoid dipoles on the opposite side of the lateral fissure (a common problem in any MEG source localization method) and to restrict the resulting dipole solutions to the vicinity of the foci of activation revealed by dSPM for MMN under control conditions.

Second, a known issue with the MxNE is its strong dependence on the regularization of the noise covariance matrix, such that too much regularization can result in multiple dipoles in circumscribed areas with similar source time courses as opposed to a single dipole capturing the same variance. To circumvent this issue, we set the regularization parameter (α, in the case of the mixed-norm solver implemented in the MNE-Python suite) such that only a single source (in either left AC or right AC, depending on the individual participant) would be returned. We then computed a subsequent MxNE on the residual (that is, the portion of the data not explained by the first MxNE). We proceeded in this manner until at least one source was present in each hemisphere, and we took only the first dipole found in each hemisphere as the final resulting source waveform for that participant/hemisphere.

For control conditions, we first restricted the time window of the MxNE to that which narrowly enveloped the primary component of the mismatch response (that is, the MMN). The resulting dipole locations were then used as weights to a subsequent MxNE in which the entire epoch duration was analyzed, resulting in the source waveforms shown in Fig. 2. Note that the MxNE jointly solves for all evoked time courses of interest, in this case standards, deviants, and deviants minus standards. The same analysis was performed for masked conditions, except that, in this case, we jointly estimated the following conditions: (i) virtual targets, by time-locking the MEG signal to points at which target standards and deviants would have occurred had they been present; (ii) undetected standards, undetected deviants, and their subtraction; and (iii) detected standards, detected deviants, and their subtraction.

Source data from each of the 20 individual participants were transformed onto a template brain (the FreeSurfer average surface) using a 2D spherical registration method that is known to yield accurate alignment of both functional and anatomical brain areas across individuals (*77*). This was performed for both the distributed dSPM and the sparse mixed-norm estimates, resulting in (i) a grand-average source map (in the case of transformed dSPMs) and (ii) a collation of dipole locations (in the case of transformed MxNEs).

In addition to the MMN-focused source analysis described above, we performed a similar analysis for the target-elicited P1 component and masker-elicited P1/N1 components, with the only difference being the source spaces used. Consistent with typical P1 source topographies (*78*), for the target-elicited P1, we used a more posterior source space defined by the transverse temporal gyrus and the adjacent portion of the planum temporale (PT) (fig. S6A). For the masker-elicited P1 and N1 components, two subanalyses were carried out—one using the same posterior source space (fig. S6A) and the other using the more anterior source space defined by the control-condition MMN (fig. S6B). This was performed for two reasons. First, although the predominant generators of both the P1 (as mentioned above) and the N1 are typically found in TTG or PT (*78*), anterior subcomponents of N1 have also been observed. Second, because this analysis was used to examine whether our masked MMN results were influenced by attention, we also wanted to be sure to use as similar a source configuration as possible for it and the masker-elicited P1/N1 components.

### Statistical analysis

All data were statistically evaluated using a repeated-measures ANOVA framework in R (http://www.r-project.org/).

For behavioral data, true- and false-positive rates were evaluated using a two-way ANOVA with response (hits versus false alarms) and tone position (10 levels) as factors. True-positive (that is, hit) rates were further evaluated after they had been sorted into their respective frequency bins using a two-way ANOVA with frequency (8 levels) and tone position (10 levels) as factors.

For neural responses under control conditions, we compared the amplitudes of the responses elicited by both standards and deviants in both left AC and right AC. Quantified amplitudes were defined as follows. First, within each participant, the average responses for standards and deviants were combined into a single grand-average response, separately for each hemisphere. We then identified the negative-going peak (occurring between 100 and 200 ms) of this grand-average waveform. The amplitude values for deviants and standards were defined as the average amplitude (per condition) within 50 ms (±25) of the latency of the grand-average peak. These amplitude values were entered into a two-way ANOVA with condition (standards versus deviants) and hemisphere as factors.

For neural responses under masked conditions, we compared the amplitudes of the responses elicited by both standards and deviants in both left AC and right AC for both detected and undetected standard streams, a three-factor design. Quantified amplitudes were defined as the average amplitude between 100 and 250 ms (separately for each hemisphere), reflecting the slightly later/broader nature of MMN under masked conditions. The same pattern of results held when we used the peak-picking procedure from the control conditions (that is, finding the negative-going peak of the grand-average between 100 and 200 ms and taking the average amplitude within 50 ms of the latency of that peak, per condition). These amplitude values were entered into a three-way ANOVA with condition (standards versus deviants), percept (detected versus undetected), and hemisphere as factors. In addition, to further evaluate the effect of standard-stream perception on MMN generation, we directly compared MMN amplitudes for detected and undetected standard streams using a two-way ANOVA with percept (detected versus undetected) and hemisphere (left versus right) as factors. MMN amplitude was defined as for the individual conditions but using the deviant-minus-standard difference waveforms.

### Bayes factor analysis

Because we were interested in whether the MMN would be elicited in the context of unperceived standard streams, we further analyzed MMN amplitudes in the context of unperceived standard streams using Bayes factors (*13*). With the mean and SE of an effect of interest (in our case, MMN amplitude in the context of unperceived standard streams), as well as a specification of a population effect-size “plausibility” distribution, the Bayes factor (ranging between zero and ∞) gives an indication of whether statistically nonsignificant differences actually provide evidence for the null hypothesis rather than being merely insensitive to a potential effect. Despite being a continuous metric, values less than ^1^/_3_ are conventionally taken as “substantial” evidence in favor of the null hypothesis, whereas values between ^1^/_3_ and 1 are taken as weak or “anecdotal” evidence (*79*). Values greater than 1 (3) are taken as weak (substantial) evidence against the null hypothesis.

To compute Bayes factors, we used the online calculator provided by Dienes (*80*), which allows one to specify (in addition to the required mean and SE of the effect of interest) the shape of the plausibility distribution for the alternative hypothesis (here, that the MMN *was* elicited in the context of unperceived standard streams). We first used a uniform distribution with a minimum of zero and a maximum of the mean MMN amplitude observed in the context of perceived standard streams. Intuitively, this indicates that although we did not precisely predict what the amplitude of the population mean MMN during unperceived standard streams would be, we assumed that it would be negative and that it could not be larger than that observed in the context of perceived standard streams. Second, because we wondered whether a uniform plausibility distribution might be too “vague” (that is, one might predict that MMN in the context of unperceived standard streams, even if present, would likely be smaller than that in the context of perceived standard streams), we also used a half-normal distribution with a mode of zero and an SD of half of the perceived-context MMN amplitude. As for the uniform specification, this assumes that the effective maximum plausible population effect size would be that which we observed in the context of perceived standard streams (that is, 2 SDs from the mode). However, it is a more conservative specification because values closer to zero are taken to be more plausible than those farther from it.

## SUPPLEMENTARY MATERIALS

Supplementary material for this article is available at http://advances.sciencemag.org/cgi/content/full/1/10/e1500677/DC1 Fig. S1. Neural activity under masked conditions after equalization of the number of epochs in each frequency bin across detected and undetected standards and deviants. Fig. S2. Neural activity under control conditions, binned by frequency. Fig. S3. Neural activity under masked conditions after epoch equalization, with emphasis on the P1 latency range. Fig. S4. Masker-elicited P1 responses using the same source space as was used for MMN. Fig. S5. Masker-elicited N1 responses using the same source space as was used for MMN. Fig. S6. Source-space vertices for P1 and MMN source analyses. Table S1. Target-tone P1 statistics. Table S2. Masker-tone P1/N1 statistics. Audio S1. Example of an auditory oddball sequence (an isochronous 902-Hz standard stream with a 947-Hz deviant and a 500-ms stimulus onset asynchrony) embedded in a random multitone masker cloud. Audio S2. Example of an auditory oddball sequence in isolation.

## References

[1] Baars B. J., Franklin S., How conscious experience and working memory interact. Trends Cogn. Sci. 7, 166–172 (2003). 12691765 10.1016/s1364-6613(03)00056-1

[2] Lamme V. A. F., Why visual attention and awareness are different. Trends Cogn. Sci. 7, 12–18 (2003). 12517353 10.1016/s1364-6613(02)00013-x

[3] A. Gilchrist, N. Cowan, in *Unconscious Memory Representations in Perception: Processes and Mechanisms in the Brain*, I. Czigler, I. Winkler, Eds. (John Benjamins B.V., Amsterdam, 2010), pp. 1–35.

[4] Näätänen R., Gaillard A. W. K., Mäntysalo S., Early selective-attention effect on evoked potential reinterpreted. Acta Psychol. 42, 313–329 (1978). 10.1016/0001-6918(78)90006-9 685709

[5] Hari R., Hämäläinen M., Ilmoniemi R., Kaukoranta E., Reinikainen K., Salminen J., Alho K., Näätänen R., Sams M., Responses of the primary auditory cortex to pitch changes in a sequence of tone pips: Neuromagnetic recordings in man. Neurosci. Lett. 50, 127–132 (1984). 6493619 10.1016/0304-3940(84)90474-9

[6] Squires N. K., Squires K. C., Hillyard S. A., Two varieties of long-latency positive waves evoked by unpredictable auditory stimuli in man. Electroencephalogr. Clin. Neurophysiol. 38, 387–401 (1975). 46819 10.1016/0013-4694(75)90263-1

[7] Näätänen R., Kujala T., Winkler I., Auditory processing that leads to conscious perception: A unique window to central auditory processing opened by the mismatch negativity and related responses. Psychophysiology 48, 4–22 (2011). 20880261 10.1111/j.1469-8986.2010.01114.x

[8] Sussman E. S., Chen S., Sussman-Fort J., Dinces E., The five myths of MMN: Redefining how to use MMN in basic and clinical research. Brain Topogr. 27, 553–564 (2014). 24158725 10.1007/s10548-013-0326-6 PMC4000291

[9] Chennu S., Bekinschtein T. A., Arousal modulates auditory attention and awareness: Insights from sleep, sedation, and disorders of consciousness. Front. Psychol. 3, 65 (2012). 22403565 10.3389/fpsyg.2012.00065 PMC3293189

[10] Morlet D., Fischer C., MMN and novelty P3 in coma and other altered states of consciousness: A review. Brain Topogr. 27, 467–479 (2014). 24281786 10.1007/s10548-013-0335-5 PMC5034015

[11] G. Kidd, C. R. Mason, V. M. Richards, F. J. Gallun, N. I. Durlach, in *Auditory Perception of Sound Sources*, W. A. Yost, A. N. Popper, R. R. Fay, Eds. (Springer, New York, 2008), pp. 143–190.

[12] Gutschalk A., Micheyl C., Oxenham A. J., Neural correlates of auditory perceptual awareness under informational masking. PLOS Biol. 6, e138 (2008). 18547141 10.1371/journal.pbio.0060138 PMC2422852

[13] Dienes Z., Using Bayes to get the most out of non-significant results. Front. Psychol. 5, 781 (2014). 25120503 10.3389/fpsyg.2014.00781 PMC4114196

[14] Woldorff M. G., Gallen C. C., Hampson S. A., Hillyard S. A., Pantev C., Sobel D., Bloom F. E., Modulation of early sensory processing in human auditory cortex during auditory selective attention. Proc. Natl. Acad. Sci. U.S.A. 90, 8722–8726 (1993). 8378354 10.1073/pnas.90.18.8722 PMC47430

[15] Sams M., Paavilainen P., Alho K., Näätänen R., Auditory frequency discrimination and event-related potentials. Electroencephalogr. Clin. Neurophysiol. 62, 437–448 (1985). 2415340 10.1016/0168-5597(85)90054-1

[16] Tiitinen H., May P., Reinikainen K., Näätänen R., Attentive novelty detection in humans is governed by pre-attentive sensory memory. Nature 372, 90–92 (1994). 7969425 10.1038/372090a0

[17] Demany L., Ramos C., On the binding of successive sounds: Perceiving shifts in nonperceived pitches. J. Acoust. Soc. Am. 117, 833–841 (2005). 15759703 10.1121/1.1850209

[18] Cowan N., Winkler I., Teder W., Näätänen R., Memory prerequisites of mismatch negativity in the auditory event-related potential (ERP). J. Exp. Psychol. Learn. Mem. Cogn. 19, 909–921 (1993). 8345328 10.1037//0278-7393.19.4.909

[19] Snyder J. S., Gregg M. K., Weintraub D. M., Alain C., Attention, awareness, and the perception of auditory scenes. Front. Psychol. 3, 15 (2012). 22347201 10.3389/fpsyg.2012.00015 PMC3273855

[20] Woldorff M. G., Hackley S. A., Hillyard S. A., The effects of channel-selective attention on the mismatch negativity wave elicited by deviant tones. Psychophysiology 28, 30–42 (1991). 1886962 10.1111/j.1469-8986.1991.tb03384.x

[21] Alain C., Woods D. L., Attention modulates auditory pattern memory as indexed by event-related brain potentials. Psychophysiology 34, 534–546 (1997). 9299908 10.1111/j.1469-8986.1997.tb01740.x

[22] Sussman E., Ritter W., Vaughan H. G. Jr, Attention affects the organization of auditory input associated with the mismatch negativity system. Brain Res. 789, 130–138 (1998). 9602095 10.1016/s0006-8993(97)01443-1

[23] Sussman E., Winkler I., Wang W., MMN and attention: Competition for deviance detection. Psychophysiology 40, 430–435 (2003). 12946116 10.1111/1469-8986.00045

[24] Näätänen R., Paavilainen P., Tiitinen H., Jiang D., Alho K., Attention and mismatch negativity. Psychophysiology 30, 436–450 (1993). 8416070 10.1111/j.1469-8986.1993.tb02067.x

[25] Pulvermüller F., Shtyrov Y., Language outside the focus of attention: The mismatch negativity as a tool for studying higher cognitive processes. Prog. Neurobiol. 79, 49–71 (2006). 16814448 10.1016/j.pneurobio.2006.04.004

[26] Szalárdy O., Bőhm T. M., Bendixen A., Winkler I., Event-related potential correlates of sound organization: Early sensory and late cognitive effects. Biol. Psychol. 93, 97–104 (2013). 23384511 10.1016/j.biopsycho.2013.01.015

[27] Näätänen R., Alho K., Generators of electrical and magnetic mismatch responses in humans. Brain Topogr. 7, 315–320 (1995). 7577329 10.1007/BF01195257

[28] Gutschalk A., Dykstra A. R., Functional imaging of auditory scene analysis. Hear. Res. 307, 98–110 (2014). 23968821 10.1016/j.heares.2013.08.003

[29] Liégeois-Chauvel C., Musolino A., Badier J. M., Marquis P., Chauvel P., Evoked potentials recorded from the auditory cortex in man: Evaluation and topography of the middle latency components. Electroencephalogr. Clin. Neurophysiol. 92, 204–214 (1994). 7514990 10.1016/0168-5597(94)90064-7

[30] Halgren E., Baudena P., Clarke J. M., Heit G., Liégeois C., Chauvel P., Musolinoa A., Intracerebral potentials to rare target and distractor auditory and visual stimuli. I. Superior temporal plane and parietal lobe. Electroencephalogr. Clin. Neurophysiol. 94, 191–220 (1995). 7536154 10.1016/0013-4694(94)00259-n

[31] Ulanovsky N., Las L., Nelken I., Processing of low-probability sounds by cortical neurons. Nat. Neurosci. 6, 391–398 (2003). 12652303 10.1038/nn1032

[32] Fishman Y. I., Steinschneider M., Searching for the mismatch negativity in primary auditory cortex of the awake monkey: Deviance detection or stimulus specific adaptation? J. Neurosci. 32, 15747–15758 (2012). 23136414 10.1523/JNEUROSCI.2835-12.2012 PMC3641775

[33] Giard M.-H., Perrin F., Pernier J., Bouchet P., Brain generators implicated in the processing of auditory stimulus deviance: A topographic event-related potential study. Psychophysiology 27, 627–640 (1990). 2100348 10.1111/j.1469-8986.1990.tb03184.x

[34] Alho K., Woods D. L., Algazi A., Knight R. T., Näätänen R., Lesions of frontal cortex diminish the auditory mismatch negativity. Electroencephalogr. Clin. Neurophysiol. 91, 353–362 (1994). 7525232 10.1016/0013-4694(94)00173-1

[35] Garrido M. I., Friston K. J., Kiebel S. J., Stephan K. E., Baldeweg T., Kilner J. M., The functional anatomy of the MMN: A DCM study of the roving paradigm. Neuroimage 42, 936–944 (2008). 18602841 10.1016/j.neuroimage.2008.05.018 PMC2640481

[36] Wacongne C., Changeux J.-P., Dehaene S., A neuronal model of predictive coding accounting for the mismatch negativity. J. Neurosci. 32, 3665–3678 (2012). 22423089 10.1523/JNEUROSCI.5003-11.2012 PMC6703454

[37] Deouell L. Y., The frontal generator of the mismatch negativity revisited. *J. Psychophysiol.* 21, 188–203 (2007).

[38] Dehaene S., Charles L., King J.-R., Marti S., Toward a computational theory of conscious processing. Curr. Opin. Neurobiol. 25, 76–84 (2014). 24709604 10.1016/j.conb.2013.12.005 PMC5635963

[39] Grimm S., Escera C., Auditory deviance detection revisited: Evidence for a hierarchical novelty system. Int. J. Psychophysiol. 85, 88–92 (2012). 21669238 10.1016/j.ijpsycho.2011.05.012

[40] Königs L., Gutschalk A., Functional lateralization in auditory cortex under informational masking and in silence. Eur. J. Neurosci. 36, 3283–3290 (2012). 22817639 10.1111/j.1460-9568.2012.08240.x

[41] Gutschalk A., Micheyl C., Melcher J. R., Rupp A., Scherg M., Oxenham A. J., Neuromagnetic correlates of streaming in human auditory cortex. J. Neurosci. 25, 5382–5388 (2005). 15930387 10.1523/JNEUROSCI.0347-05.2005 PMC1237040

[42] Gutschalk A., Rupp A., Dykstra A. R., Interaction of streaming and attention in human auditory cortex. PLOS One 10, e0118962 (2015). 25785997 10.1371/journal.pone.0118962 PMC4364770

[43] May P. J. C., Tiitinen H., Mismatch negativity (MMN), the deviance-elicited auditory deflection, explained. Psychophysiology 47, 66–122 (2010). 19686538 10.1111/j.1469-8986.2009.00856.x

[44] Jääskeläinen I. P., Ahveninen J., Bonmassar G., Dale A. M., Ilmoniemi R. J., Levänen S., Lin F.-H., May P., Melcher J., Stufflebeam S., Tiitinen H., Belliveau J. W., Human posterior auditory cortex gates novel sounds to consciousness. Proc. Natl. Acad. Sci. U.S.A. 101, 6809–6814 (2004). 15096618 10.1073/pnas.0303760101 PMC404127

[45] Kauramäki J., Jääskeläinen I. P., Sams M., Selective attention increases both gain and feature selectivity of the human auditory cortex. PLOS One 2, e909 (2007). 17878944 10.1371/journal.pone.0000909 PMC1975472

[46] Ahveninen J., Hämäläinen M., Jääskeläinen I. P., Ahlfors S. P., Huang S., Lin F.-H., Raij T., Sams M., Vasios C. E., Belliveau J. W., Attention-driven auditory cortex short-term plasticity helps segregate relevant sounds from noise. Proc. Natl. Acad. Sci. U.S.A. 108, 4182–4187 (2011). 21368107 10.1073/pnas.1016134108 PMC3053977

[47] Strauss M., Sitt J. D., King J.-R., Elbaz M., Azizi L., Buiatti M., Naccache L., van Wassenhove V., Dehaene S., Disruption of hierarchical predictive coding during sleep. Proc. Natl. Acad. Sci. U.S.A. 112, E1353–E1362 (2015). 25737555 10.1073/pnas.1501026112 PMC4371991

[48] Bekinschtein T. A., Dehaene S., Rohaut B., Tadel F., Cohen L., Naccache L., Neural signature of the conscious processing of auditory regularities. Proc. Natl. Acad. Sci. U.S.A. 106, 1672–1677 (2009). 19164526 10.1073/pnas.0809667106 PMC2635770

[49] Loewy D. H., Campbell K. B., Bastien C., The mismatch negativity to frequency deviant stimuli during natural sleep. Electroencephalogr. Clin. Neurophysiol. 98, 493–501 (1996). 8763509 10.1016/0013-4694(96)95553-4

[50] Atienza M., Cantero J. L., Dominguez-Marin E., Mismatch negativity (MMN): An objective measure of sensory memory and long-lasting memories during sleep. Int. J. Psychophysiol. 46, 215–225 (2002). 12445949 10.1016/s0167-8760(02)00113-7

[51] Yppärilä H., Karhu J., Westerén-Punnonen S., Musialowicz T., Partanen J., Evidence of auditory processing during postoperative propofol sedation. Clin. Neurophysiol. 113, 1357–1364 (2002). 12140017 10.1016/s1388-2457(02)00158-x

[52] Koelsch S., Heinke W., Sammler D., Olthoff D., Auditory processing during deep propofol sedation and recovery from unconsciousness. Clin. Neurophysiol. 117, 1746–1759 (2006). 16807099 10.1016/j.clinph.2006.05.009

[53] Price L. J., Kremen I., Variations in behavioral response threshold within the REM period of human sleep. Psychophysiology 17, 133–140 (1980). 7375615 10.1111/j.1469-8986.1980.tb00125.x

[54] Burton S. A., Harsh J. R., Badia P., Cognitive activity in sleep and responsiveness to external stimuli. Sleep 11, 61–68 (1988). 3363271

[55] Kouider S., Andrillon T., Barbosa L. S., Goupil L., Bekinschtein T. A., Inducing task-relevant responses to speech in the sleeping brain. Curr. Biol. 24, 2208–2214 (2014). 25220055 10.1016/j.cub.2014.08.016 PMC4175175

[56] Ibáñez A. M., Martín R. S., Hurtado E., López V., ERPs studies of cognitive processing during sleep. Int. J. Psychol. 44, 290–304 (2009). 22029558 10.1080/00207590802194234

[57] Kotchoubey B., Event-related potential measures of consciousness: Two equations with three unknowns. Prog. Brain Res. 150, 427–444 (2005). 16186040 10.1016/S0079-6123(05)50030-X

[58] Sallinen M., Kaartinen J., Lyytinen H., Processing of auditory stimuli during tonic and phasic periods of REM sleep as revealed by event-related brain potentials. J. Sleep Res. 5, 220–228 (1996). 9065873 10.1111/j.1365-2869.1996.00220.x

[59] Heinke W., Kenntner R., Gunter T. C., Sammler D., Olthoff D., Koelsch S., Sequential effects of increasing propofol sedation on frontal and temporal cortices as indexed by auditory event-related potentials. Anesthesiology 100, 617–625 (2004). 15108977 10.1097/00000542-200403000-00023

[60] Simpson T. P., Manara A. R., Kane N. M., Barton R. L., Rowlands C. A., Butler S. R., Effect of propofol anaesthesia on the event-related potential mismatch negativity and the auditory-evoked potential N1. Br. J. Anaesth. 89, 382–388 (2002). 12402715

[61] Y. Shtyrov, F. Pulvermüller, in *Unconscious Memory Representations in Perception: Processes and Mechanisms in the Brain*, I. Czigler, I. Winkler, Eds. (John Benjamins B.V., Amsterdam, 2010), pp. 179–207.

[62] Davis M. H., Coleman M. R., Absalom A. R., Rodd J. M., Johnsrude I. S., Matta B. F., Owen A. M., Menon D. K., Dissociating speech perception and comprehension at reduced levels of awareness. Proc. Natl. Acad. Sci. U.S.A. 104, 16032–16037 (2007). 17938125 10.1073/pnas.0701309104 PMC2042157

[63] Wijnen V. J. M., van Boxtel G. J. M., Eilander H. J., de Gelder B., Mismatch negativity predicts recovery from the vegetative state. Clin. Neurophysiol. 118, 597–605 (2007). 17239656 10.1016/j.clinph.2006.11.020

[64] Kotchoubey B., Lang S., Mezger G., Schmalohr D., Schneck M., Semmler A., Bostanov V., Birbaumer N., Information processing in severe disorders of consciousness: Vegetative state and minimally conscious state. Clin. Neurophysiol. 116, 2441–2453 (2005). 16002333 10.1016/j.clinph.2005.03.028

[65] Tzovara A., Rossetti A. O., Spierer L., Grivel J., Murray M. M., Oddo M., De Lucia M., Progression of auditory discrimination based on neural decoding predicts awakening from coma. Brain 136, 81–89 (2013). 23148350 10.1093/brain/aws264

[66] Elhilali M., Xiang J., Shamma S. A., Simon J. Z., Interaction between attention and bottom-up saliency mediates the representation of foreground and background in an auditory scene. PLOS Biol. 7, e1000129 (2009). 19529760 10.1371/journal.pbio.1000129 PMC2690434

[67] D. M. Green, J. A. Swets, *Signal Detection Theory and Psychophysics* (Wiley, New York, 1966).

[68] Uusitalo M. A., Ilmoniemi R. J., Signal-space projection method for separating MEG or EEG into components. Med. Biol. Eng. Comput. 35, 135–140 (1997). 9136207 10.1007/BF02534144

[69] Gramfort A., Luessi M., Larson E., Engemann D. A., Strohmeier D., Brodbeck C., Parkkonen L., Hämäläinen M. S., MNE software for processing MEG and EEG data. Neuroimage 86, 446–460 (2014). 24161808 10.1016/j.neuroimage.2013.10.027 PMC3930851

[70] Dale A. M., Fischl B., Sereno M. I., Cortical surface-based analysis: I. Segmentation and surface reconstruction. Neuroimage 9, 179–194 (1999). 9931268 10.1006/nimg.1998.0395

[71] Fischl B., Sereno M. I., Dale A. M., Cortical surface-based analysis: II. Inflation, flattening, and a surface-based coordinate system. Neuroimage 9, 195–207 (1999). 9931269 10.1006/nimg.1998.0396

[72] Besl P. J., McKay N. D., A method for registration of 3-D shapes. IEEE Trans. Pattern Anal. Mach. Intell. 14, 239–256 (1992).

[73] Hämäläinen M. S., Ilmoniemi R. J., Interpreting magnetic fields of the brain: Minimum norm estimates. Med. Biol. Eng. Comput. 32, 35–42 (1994). 8182960 10.1007/BF02512476

[74] Dale A. M., Sereno M. I., Improved localizadon of cortical activity by combining EEG and MEG with MRI cortical surface reconstruction: A linear approach. J. Cogn. Neurosci. 5, 162–176 (1993). 23972151 10.1162/jocn.1993.5.2.162

[75] Dale A. M., Liu A. K., Fischl B. R., Buckner R. L., Belliveau J. W., Lewine J. D., Halgren E., Dynamic statistical parametric mapping: Combining fMRI and MEG for high-resolution imaging of cortical activity. Neuron 26, 55–67 (2000). 10798392 10.1016/s0896-6273(00)81138-1

[76] Gramfort A., Kowalski M., Hämäläinen M., Mixed-norm estimates for the M/EEG inverse problem using accelerated gradient methods. Phys. Med. Biol. 57, 1937–1961 (2012). 22421459 10.1088/0031-9155/57/7/1937 PMC3566429

[77] Fischl B., Sereno M. I., Tootell R. B., Dale A. M., High-resolution intersubject averaging and a coordinate system for the cortical surface. Hum. Brain Mapp. 8, 272–284 (1999). 10619420 10.1002/(SICI)1097-0193(1999)8:4<272::AID-HBM10>3.0.CO;2-4 PMC6873338

[78] A. Gutschalk, in *Magnetoencephalography: From Signals to Dynamic Cortical Networks*, S. Supek, C. J. Aine, Eds. (Springer-Verlag, Berlin, 2014), pp. 679–711.

[79] H. Jeffreys, *Theory of Probability* (Oxford Univ. Press, Oxford, ed. 2, 1948).

[80] Z. Dienes, Calculate your Bayes factor, http://www.lifesci.sussex.ac.uk/home/Zoltan_Dienes/inference/bayes_factor.swf.

